# ATG9B is a tissue-specific homotrimeric lipid scramblase that can compensate for ATG9A

**DOI:** 10.1080/15548627.2023.2275905

**Published:** 2023-11-08

**Authors:** George N. Chiduza, Acely Garza-Garcia, Eugenia Almacellas, Stefano De Tito, Valerie E Pye, Alexander R. van Vliet, Peter Cherepanov, Sharon A. Tooze

**Affiliations:** a Molecular Cell Biology of Autophagy, The Francis Crick Institute, London, UK; b Mycobacterial Metabolism and Antibiotic Research Laboratory, The Francis Crick Institute, London, UK; c Chromatin Structure and Mobile DNA Laboratory, The Francis Crick Institute, London, UK; d Department of Infectious Disease, Imperial College London, London, UK

**Keywords:** ATG9A, autophagy, cryo-EM, membrane protein dynamics, phagophore, scramblase

## Abstract

Macroautophagy/autophagy is a fundamental aspect of eukaryotic biology, and the autophagy-related protein ATG9A is part of the core machinery facilitating this process. In addition to ATG9A vertebrates encode ATG9B, a poorly characterized paralog expressed in a subset of tissues. Herein, we characterize the structure of human ATG9B revealing the conserved homotrimeric quaternary structure and explore the conformational dynamics of the protein. Consistent with the experimental structure and computational chemistry, we establish that ATG9B is a functional lipid scramblase. We show that ATG9B can compensate for the absence of ATG9A in starvation-induced autophagy displaying similar subcellular trafficking and steady-state localization. Finally, we demonstrate that ATG9B can form a heteromeric complex with ATG2A. By establishing the molecular structure and function of ATG9B, our results inform the exploration of niche roles for autophagy machinery in more complex eukaryotes and reveal insights relevant across species.

**Abbreviation:** ATG: autophagy related; CHS: cholesteryl hemisuccinate; cryo-EM: single-particle cryogenic electron microscopy; CTF: contrast transfer function: CTH: C- terminal α helix; FSC: fourier shell correlation; HDIR: HORMA domain interacting region; LMNG: lauryl maltose neopentyl glycol; MD: molecular dynamics simulations; MSA: multiple sequence alignment; NBD-PE: 1,2-dioleoyl-sn-glycero-3-phosphoethanolamine-N-(7-nitro-2–1,3-benzoxadiazol-4-yl ammonium salt); POPC: palmitoyl-2-oleoyl-sn-glycero-3-phosphocholine; RBG: repeating beta groove domain; RMSD: root mean square deviation; SEC: size-exclusion chromatography; TMH: transmembrane helix

## Introduction

An autophagosome is a double-membrane organelle formed during autophagy to deliver a portion of cytoplasm to the lysosome for degradation. Autophagy is increased in response to cellular stresses such as nutrient starvation. During the process of macroautophagy, here called autophagy, a small phagophore is nucleated at an omegasome on the endoplasmic reticulum (ER) [1] and grows through the rapid recruitment of proteins and lipids into a mature autophagosome [2,3]. Autophagy is choreographed by a core group of proteins conserved across eukaryotes, the ATG (autophagy related) proteins. Two of them, ATG2A and ATG9A, collaborate to facilitate direct transfer of lipids to the nascent autophagosome [4–6].

ATG2A is a peripheral membrane protein that belongs to a novel class of repeating beta groove domain (RBG) proteins that can bridge separate bilayers allowing lipid flow between their outer leaflets [5,7]. There are currently no high-resolution structures of ATG2A, likely due to its conformational flexibility, however the predicted atomic model for the protein is consistent with the available low-resolution structures and data from structural mass spectrometry [8]. In this model, the RBG is a hydrophobic grove that can potentially accommodate lipid acyl chains, lowering the entropic cost of transferring lipids across the aqueous cytosol [5]. ATG9A is a lipid scramblase, that facilitates equilibration of lipids across the leaflets of a lipid bilayer. Sequence and structural homology suggest the novel ATG9 fold is evolutionarily related to type I ATP binding cassette exporters [6]. ATG9A forms obligate trimers through domain swapping of transmembrane domains, forming a pore at the center of the trimer that is open to the cytosol, and connected to lateral pores/branches in each of the protomers. The lateral branches are open to the cytosol and the outer leaflet of the membrane, and in the structures of ATG9A they are occupied by detergent or lipid molecules depending on whether the structures were determined in detergent micelles or in lipid nanodiscs, respectively. Using *in vitro* scramblase assays, and mutagenesis of residues in the lateral branch and central pore, in combination with molecular dynamics simulations (MD) in a lipid bilayer, these pores have been implicated in lipid scrambling, although the precise mechanism remains to be fully elucidated [6,9].

An emerging paradigm is the coupling of RBG lipid transfer proteins to lipid scramblases on either membrane to resolve bilayer asymmetry in source and target membranes that would arise from their lipid transfer activity [10,11]. Through its C terminus ATG2A interacts with ATG9A in a heterotetrameric complex providing a mechanism to couple the lipid transfer and scramblase activities which is essential for autophagosome biogenesis. The central pore and lateral branches of ATG9A are also connected to the cytosol through perpendicular branches formed at the protomer interfaces. Integrative modeling of the ATG9A-ATG2A complex suggested that these branches may facilitate direct lipid hand over between ATG9A and ATG2A [8]. TMEM41B and VMP1 are ER resident lipid scramblases that interact with the N terminus of ATG2A and are thought, in concert with ATG9A to form the core lipid transfer complex at ER-phagophore contacts sites, where presumably local lipid synthesis in the ER thermodynamically drives lipid flow through the complex into the elongating phagophore [12–14].

ATG9A localizes to the trans-Golgi network and endosomal compartments from where, under nutrient starvation, it re-localizes in small vesicles around omegasomes. This dispersal from the trans-Golgi network and endosomal compartment to the sites of autophagy nucleation is essential for autophagosome biogenesis [15–17]. The dispersed ATG9A compartment dynamically interacts with and delivers other autophagy effectors to the omegasome [16,17]. It has also been speculated that ATG9A vesicles are the seeding membrane of the nascent phagophore, as shown in yeast and is consistent with the ATG9A-ATG2A-TMEM41B-VMP1 lipid scramblase-lipid transfer protein partnership model described above [12,18,19]. However, this is controversial since ATG9A could not be detected in the mammalian phagophore [16,20].

Although ATG9A is referred to as the only integral membrane protein among the ATG proteins, ATG9B was discovered and reported simultaneously with ATG9A [21]. ATG9B is described as a vertebrate specific paralog and, in contrast to the ubiquitously expressed ATG9A, is enriched in the placenta and pituitary gland suggesting a specialized role (https://www.proteinatlas.org/) [22]. ATG9B was shown to play a role in autophagy, has been implicated in tumorigenesis, particularly hepatocellular carcinoma and, has been proposed as a target for autophagy inhibition in gliomas, as well as other pathologies [23–26]. Its importance notwithstanding, the molecular structure and functions of ATG9B, as well as its cellular localization and trafficking have not been studied to the same extent as ATG9A. Here, we present a structure of human ATG9B from single-particle cryogenic electron microscopy (cryo-EM) and conformational heterogeneity analysis from these data that inform the understanding of the molecular mechanisms not only of ATG9B but ATG9 domain proteins generally. We demonstrate that ATG9B is a functional lipid scramblase and report a preliminary analysis of ATG9B cellular localization and trafficking.

## Results

### Bioinformatic analyses of ATG9B

**Figure 1.**
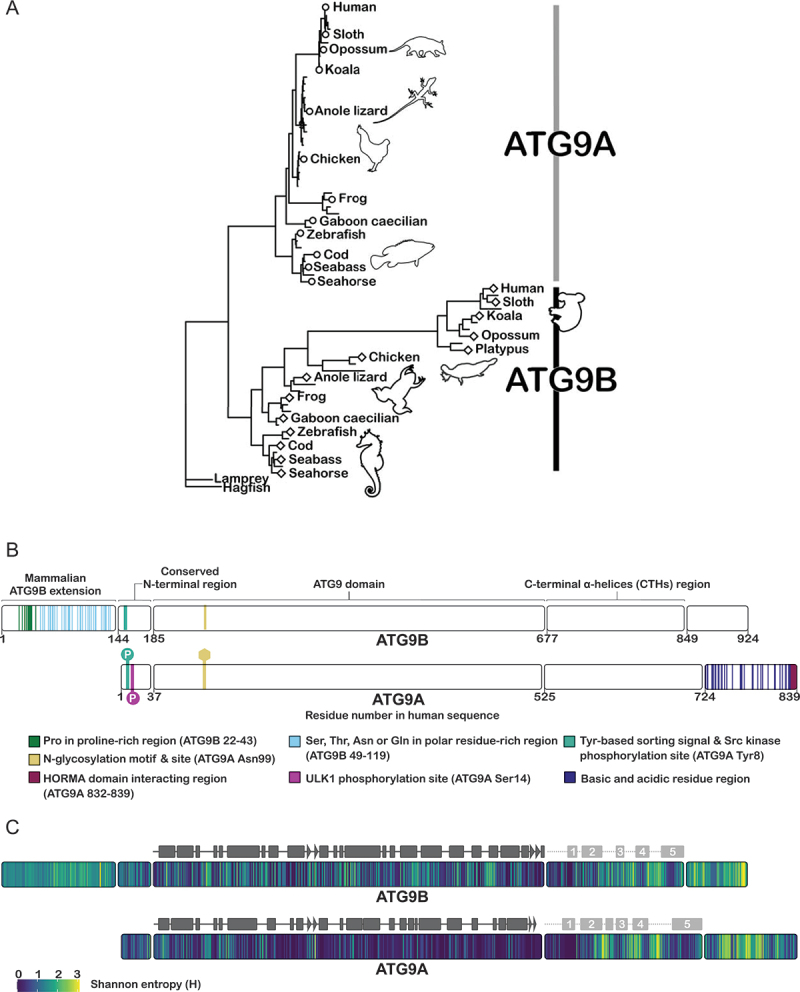
Sequence architecture, variability and evolution of vertebrate ATG9A and ATG9B. (A) phylogenetic tree estimated from a multiple sequence alignment of the ATG9 domain of ATG9A and ATG9B from 71 Chordata species. See table 1 for species names, and accession numbers. (B) sequence features, motifs, domain boundaries and selected posttranslational modifications of human ATG9A (Q7Z3C6–1) and human ATG9B (Q674R7–1). (C) Shannon entropy values (H) obtained for the Ensembl orthologs alignments of ATG9A and of ATG9B. The experimental regular secondary structure features (α-helices in rectangles and β-strands in triangles) are shown in dark gray; in dashed lines and light gray are those predicted by AlphaFold.

To reconstruct the evolution of ATG9B and develop hypotheses for its potential molecular and cellular functions we turned to multiple protein sequence analyses. A maximum-likelihood phylogenetic tree (Figure 1A) was generated from an alignment of the ATG9 domain (Pfam domain PF04109), residues 185–676 in human ATG9B (Figure 1B), of 71 ATG9 Chordata homologs (Table 1). ATG9A and ATG9B are paralogous proteins only present in jawed vertebrates (gnathostomes). Basal vertebrates such as hagfish and lampreys have only one ATG9 gene. Interestingly, the longer branch lengths of mammalian ATG9B in the tree compared to those of ATG9A indicates that the ATG9 domain sequences of the mammalian ATG9Bs are changing faster than those of the mammalian ATG9A (Figure 1A). Shannon entropy (H value) analysis of two separate multiple sequence alignments (MSAs) of the full-length human orthologs of ATG9A and ATG9B was also performed, where a H value less than one is considered conserved and H value greater than two is considered variable [27]. In this analysis, consistent with the phylogenetic analysis, the ATG9 domains of ATG9A and ATG9B had median H values of 0.24 and 0.90 respectively (Figure 1B,C).

**Table 1.** Species names and accession numbers of ATG9 homologs used in phylogenetic analysis.

Label	Accession code	Common name	Scientific name
Anole_lizard_A	ENSACAP00000012906	Anole lizard	Anolis carolinensis
Anole_lizard_B	XP_008121259.1	Anole lizard	Anolis carolinensis
Central_bearded_dragon_B	XP_020655810.1	Central bearded dragon	Pogona vitticeps
Corn_snake_XP_034279395.1	XP_034279395.1	Corn snake	Pantherophis guttatus
Chicken_B	ENSGALP00010040139	Chicken	Gallus gallus
Japanese_quail-ENSCJPP00005007903	ENSCJPP00005007903	Japanese quail	Coturnix japonica
Zebra_finch-ENSTGUP00000022016	ENSTGUP00000022016	Zebra finch	Taeniopygia guttata
Carp-B-ENSCCRP00000029789	ENSCCRP00000029789	Common carp	Cyprinus carpio carpio
Zebrafish-ENSDARP00000120741	ENSDARP00000120741	Zebrafish	Danio rerio
Cod-ENSGMOP00000051691	ENSGMOP00000051691	Atlantic cod	Gadus morhua
Eel-ENSMAMP00000023840	ENSMAMP00000023840	Zig-zag Eel	Mastacembelus armatus
Fugu-ENSTRUP00000005297	ENSTRUP00000005297	Fugu	Takifugu rubripes
Seabass-ENSDLAP00005062378	ENSDLAP00005062378	European seabass	Dicentrarchus labrax
Seahorse-XP_019734422.1	XP_019734422.1	Tiger tail seahorse	Hippocampus comes
Coqui_frog-KAG9468555.1	KAG9468555.1	Coqui frog	Eleutherodactylus coqui
Rana_temporaria-B	XP_040211350.1	Rana temporaria	Rana temporaria
Xtropicalis_A	ENSXETP00000026208	Tropical clawed frog	Xenopus tropicalis
Geotrypetes_seraphini-XP_033787984.1	XP_033787984.1	Gaboon caecilian	Geotrypetes seraphini
Microcaecilia_unicolor-XP_030051462.1	XP_030051462.1	Microcaecilia unicolor	Microcaecilia unicolor
Armadillo_B	XP_004458326.1	Nine-banded armadillo	Dasypus novemcinctus
Sloth_B	XP_037693322.1	Sloth	Choloepus didactylus
Human_B	ATG9B_HUMAN	Human	Homo sapiens
Blue_whale_B	ENSBMSP00010019199	Blue whale	Balaenoptera musculus
Echidna_B	XP_038611400.1	Echidna	Tachyglossus aculeatus
Platypus_O	XP_028933935.1	Platypus	Ornithorhynchus anatinus
Koala_B	ENSPCIP00000007259	Koala	Phascolarctos cinereus
Wombat_B	ENSVURP00010009890	Common wombat	Vombatus ursinus
Tasmanian_devil_B	ENSSHAP00000033349	Tasmanian devil	Sarcophilus harrisii
Opossum_B	ENSMODP00000004795	Opossum	Monodelphis domestica
Hagfish	ENSEBUP00000002125	Hagfish	Eptatretus burgeri
Lamprey	ENSPMAP00000004602	Sea lamprey	Petromyzon marinus
Carp-ENSCCRP00000001116	ENSCCRP00000001116	Common carp	Cyprinus carpio carpio
Carp-ENSCCRP00000001205	ENSCCRP00000001205	Common carp	Cyprinus carpio carpio
Zebrafish ENSDARP00000065410	ENSDARP00000065410	Zebrafish	Danio rerio
Cod-ENSGMOP00000011632	ENSGMOP00000011632	Atlantic cod	Gadus morhua
Eel-ENSMAMP00000006061	ENSMAMP00000006061	Zig-zag Eel	Mastacembelus armatus
Seabass-ENSDLAP00005015764	ENSDLAP00005015764	European seabass	Dicentrarchus labrax
Fugu-ENSTRUP00000038550	ENSTRUP00000038550	Fugu	Takifugu rubripes
Seahorse-ENSHCOG00000020389	ENSHCOP00000028046	Tiger tail seahorse	Hippocampus comes
Coqui_frog-KAG9469560.1	KAG9469560.1	Coqui frog	Eleutherodactylus coqui
Rana_temporaria-A	XP_040214104.1	Rana temporaria	Rana temporaria
Dwarf_clawed_frog-KAG8432226.1	KAG8432226.1	Dwarf clawed frog	Hymenochirus boettgeri
Xtropicalis_B	ENSXETP00000098693	Tropical clawed frog	Xenopus tropicalis
Geotrypetes_seraphini_A	XP_033800758.1	Geotrypetes seraphini	Geotrypetes seraphini
Microcaecilia_unicolor_A	XP_030065876.1	Microcaecilia unicolor	Microcaecilia unicolor
Armadillo_A	XP_012384164.1	Nine-banded armadillo	Dasypus novemcinctus
Human_A	ATG9A_HUMAN	Human	Homo sapiens
Sloth_A	XP_037705646.1	Sloth	Choloepus didactylus
Blue_whale_A	ENSBMSP00010001100	Blue whale	Balaenoptera musculus
Narwal_O	ENSMMNP00015006983	Narwhal	Monodon monoceros
Koala_A	ENSPCIP00000013078	Koala	Phascolarctos cinereus
Wombat_A	ENSVURP00010000335	Common wombat	Vombatus ursinus
Opossum_A	ENSMODP00000053644	Opossum	Monodelphis domestica
Tasmanian_devil_A	ENSSHAP00000037169	Tasmanian devil	Sarcophilus harrisii
Echidna_A	XP_038599496.1	Echidna	Tachyglossus aculeatus
Cassowary_A	NXE46587.1	Cassowary	Casuarius casuarius
Ostrich_O	KFV81944.1	Ostrich	Struthio camelus australis
Flycatcher_O-cds-ENSFALT00000004408	ENSFALP00000004385	Collared flycatcher	Ficedula albicollis
Kakapo	XP_030342785.1	Kakapo	Strigops habroptila
Goose-ENSABRG00000000296_cds	ENSABRP00000000260	Pink-footed goose	Anser brachyrhynchus
Chicken_A	ENSGALP00010031034	Chicken	Gallus gallus
Central_bearded_dragon_A	ENSPVIP00000029031	Central bearded dragon	Pogona vitticeps
Eastern_fence_lizard	XP_042317609.1	Eastern fence lizard	Sceloporus undulatus
Brown-spotted_pit_viper	XP_015672179.1	Brown-spotted pit viper	Protobothrops mucrosquamatus
Burmese_python	XP_007419864.1	Burmese python	Python bivittatus
Corn_snake_XP_034289648.1	XP_034289648.1	Corn snake	Pantherophis guttatus
Komodo_dragon-XP_044294227.1	XP_044294227.1	Komodo dragon	Varanus komodoensis
Townsend’s_least_gecko	XP_048341084.1	Townsend’s least gecko	Sphaerodactylus townsendi
Gekko-XP_015280563.1	XP_015280563.1	Gekko	Gekko japonicus
Sand_lizard	XP_033016151.1	Sand lizard	Lacerta agilis
Wall_lizard_A	ENSPMRP00000028251	Wall lizard	*Podarcis muralis*

In both ATG9A and B, the ATG9 domain is flanked by predicted intrinsically disordered regions of different lengths. These regions feature sections that are conserved across paralogs as well as sections that are ortholog or taxon specific. The median H values of these regions outside the ATG9 domains of ATG9A and ATG9B were, 0.68 and 1 in the conserved N terminus region; 0.88 and 1.17 in the C-terminal α-helices region; 1.55 and 1.77 in the rest of the C terminus (Figure 1B,C). These values indicate that the *N*- and C-terminal regions of each paralog have greater variability than their corresponding ATG9 domain. In mammals, the N terminus of ATG9B is about 143 residues longer than that of ATG9A and contains regions of compositional bias, specifically a proline rich region (residues 22–43 in human ATG9B) and polar residue rich regions (residues 49–119 in human ATG9B) (Figure 1B). Residues 144–184 of human ATG9B correspond to residues 1–36 of human ATG9A. In ATG9A, this region includes the tyrosine-based sorting motif, YxxΦ (x= any residue; Φ = large hydrophobic residue), which plays a role in the trafficking of ATG9A (8-YQRL-11 in human ATG9A) by mediating interaction with the trafficking adaptor protein complexes, AP1, AP2 and AP4. This motif is conserved in ATG9B (151-YERL-154 in human ATG9B), suggesting that ATG9B may interact with the same adaptor proteins [28–31]. In human ATG9A, constitutive phosphorylation by SRC kinase of Tyr8 within this sorting motif promotes the interaction with AP1 and AP2 and can also support starvation induced ULK1 phosphorylation on Ser14 [32]. This ULK1 phosphorylation site is not conserved in ATG9B. The glycosylation motif in ATG9A (99-NHS-101 in human) is conserved in ATG9B (248-NHT-250 in human) suggesting that ATG9B may be modified by N-linked glycans. After the ATG9 domain, the C terminus of mammalian ATG9B is about 85 residues shorter than that of mammalian ATG9A but the conserved regions include putative α helices as described in the detailed structural analysis of human ATG9B below. However, the HORMA domain interacting region (HDIR) (832–839 in human) that mediates ATG9A-ATG13-ATG101 complex formation is not conserved in ATG9B (Figure 1B) [33,34].

The homology of the ATG9 domains and conservation of the tyrosine-based motif suggests that ATG9B shares a similar fold and molecular functions as ATG9A, as well as similar trafficking in cells. To test this hypothesis, we determined the molecular structure, tested for scramblase activity, and characterized the intracellular localization of human ATG9B.

### Purification and single particle cryo-EM of ATG9B

**Figure 2.**
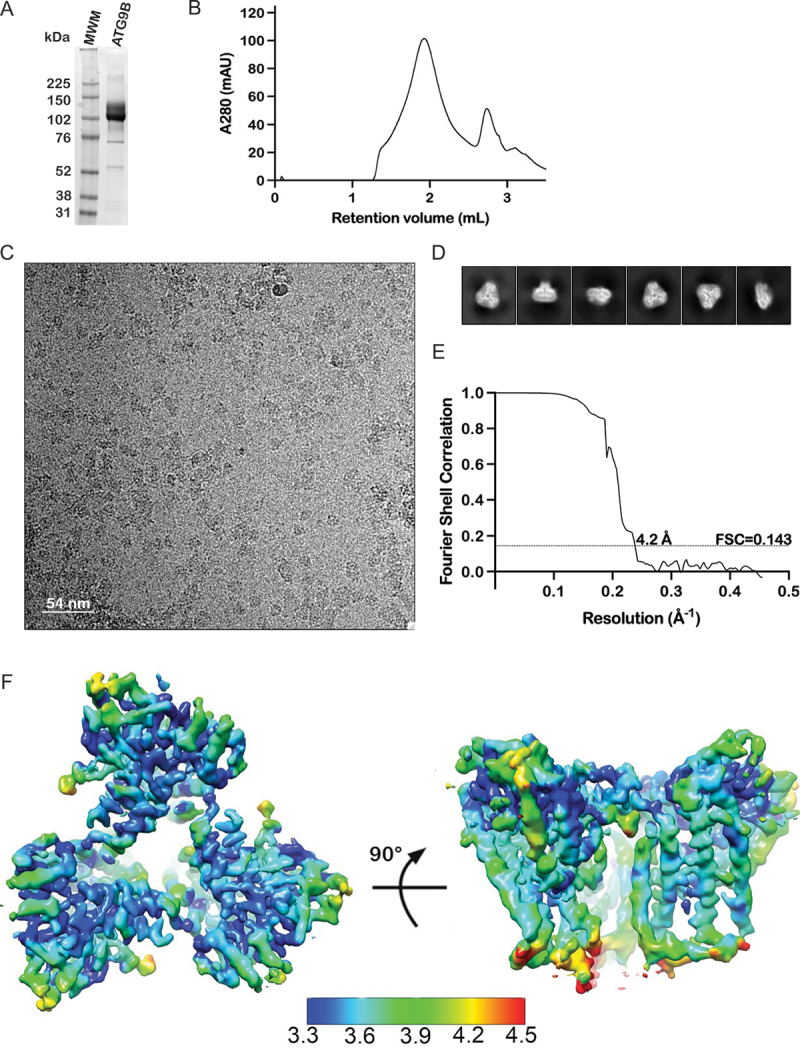
Purification and single particle cryo-EM of ATG9B. (A) Coomassie Blue stained gel of purified ATG9B (MWM = molecular weight markers) (B) size exclusion chromatogram of purified ATG9B. (C) Representative cryo-EM micrograph of purified ATG9B in vitrified ice and (D) 2D class averages of particles from these data. (E) FSC (fourier shell correlation) of the C3 symmetry imposed 3D reconstruction of ATG9B (EMD-17789). (F) C3 symmetry-imposed 3D reconstruction of ATG9B from cryo-EM colored by local resolution (Å).

**Figure 3.**
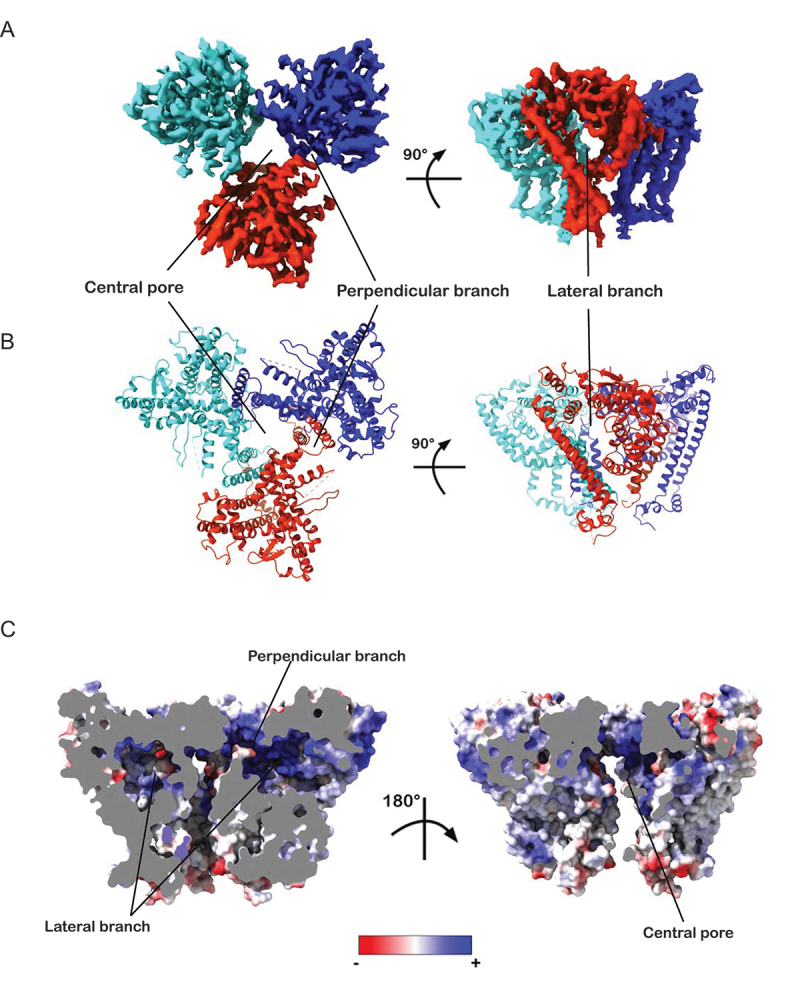
Topology and pores of ATG9B structure. (A) postprocessed cryo-EM map (C3) of ATG9B colored by protomers, except domain swapped transmembrane helices (EMD-17789). (B) ribbon representation of atomic model of ATG9B built from C3 imposed 3D reconstruction colored by protomer (PDB ID 8POE). (C) slice throughs of ATG9B isosurface colored by electrostatic potential with central pore and branches highlighted.

Full-length ATG9B, N-terminally tagged with a triple FLAG epitope, was transiently overexpressed in Expi293 cells. The protein was extracted from membranes into detergent micelles and affinity purified using anti-FLAG resin (Figure 2A). Before cryo-EM grid preparation, the sample was further purified by size-exclusion chromatography (SEC). In SEC, the affinity purified sample produced a monodisperse peak at 2.0 mL retention volume, where the ATG9A trimer elutes on the same column in these conditions [8], as well as a minor peak at 2.8 mL retention volume corresponding to 3×FLAG peptide used to elute ATG9B from the affinity resin (Figure 2B). Imaging of cryo-EM grids prepared from fractions of the SEC peak at 2 mL showed single particles consistent with the monodispersity of the protein material. 2D class averages of these particles revealed visible secondary structure features and a clear 3-fold symmetry (Figure 2C,D; Table 2). Further image processing with and without C3 symmetry imposed resulted in 3D reconstructions of ATG9B homotrimer at a global resolution of 4.2 Å (EMD-17789) and 4.6 Å (EMD-17790), respectively, while the local resolution in the core regions of the symmetry-imposed structure ranged between 3.2 and 3.8 Å (Figure 2E,F). Although imposing C3 symmetry during refinement improved overall resolution, it introduced minor artifacts in the cryo-EM map that were absent when the volume was refined without symmetry (Figure S1). Likely resulting from conformational heterogeneity, the artifacts could be partially corrected by postprocessing the reconstruction using DeepEMhancer [35], which considerably aided building of the atomistic model (Figure 3A, Movie 1, PDB ID 8POE).

**Table 2.** Cryo-EM data collection, image processing and model building.

Magnification	46,296
Voltage (kV)	300
Number of frames	32
Electron exposure (e-/Å2)	48
Defocus range (microns)	−1 to −3
Pixel size (Å)	1.08
Symmetry imposed	C3 or C1
Initial particle number	68,18,472
Final particle number	1,04,050
Map resolution (Å)	4.2(C3); 4.6(C1)
FSC threshold	0.143
*Model building*	
Starting model	ATG9B Alphafold
Chains	3
Non-hydrogen atoms	10497
Protein residues	1371
Ligands	0
*Validation*	
Molprobity clash score	9.34
Poor rotamers (%)	0
Ramachandran plot	
Favoured (%)	97.24
Allowed (%)	2.76
Disallowed (%)	0

Most of the backbone and side chains of residues corresponding to ATG9B amino acid positions 191–674 were visible in the cryo-EM maps (Figure S1E). By contrast, the *N*- and C-terminal regions were not, presumably due to conformational variability and/or intrinsic disorder (Figure 3B). ATG9B forms a homotrimer with a pore at its center connected to perpendicular branches formed at the protomer interfaces. Each protomer has a lateral branch that connects to the central pore and the perpendicular branches. These features are characteristic of the ATG9 domain structures determined to date and so is the hydrophilic nature of most of the residues lining the pores, suggesting a conserved functional mechanism (Figure 3C). Having determined a structure for ATG9B we compared it to ATG9A and explored the conformational dynamics of ATG9B in the cryo-EM dataset.

### Conformational dynamics of the ATG9B homotrimer

**Figure 4.**
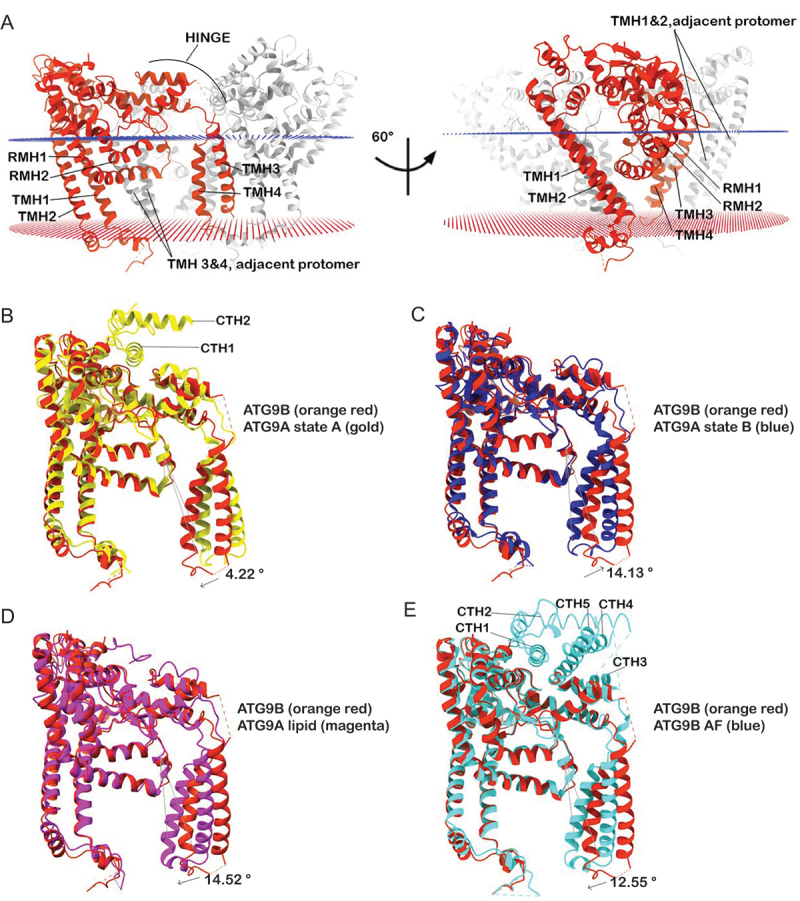
Conformational analysis of ATG9B. (A) ATG9B trimer in lipid bilayer (blue and red lattices), the transmembrane domains and HINGE region of one protomer (orange red) is highlighted. Structural alignments of ATG9B and (B) ATG9A state a (PDB ID 6WQZ), (C) ATG9A state B (PDB ID 6WR4), (D) ATG9A in nanodisc (PDB ID 7JLP) and (E) AlphaFold model of ATG9B. The faint gray vertical lines are the axis from which the relative rotations of TMH 4 to the rest of the protomer is measured between the compared structures. The arrow indicates the direction of rotation from the ATG9B structure to the compared structure and the magnitude of this rotation is indicated next to the arrow. RMH: reentrant membrane helix.

As reported for other ATG9 domain proteins [6,9,36,37], the ATG9B subunits share an extensive interaction interface within the homotrimer, with an approximately 2,494Å^2^ buried molecular surface area per subunit. The homomeric interfaces are predominantly mediated by domain swapped transmembrane helix (TMH) 3 and 4 of one protomer with TMH1 and reentrant membrane helix 1 of the adjacent protomer (Figure 4A). TMHs 3 and 4 in each protomer are connected to the rest of the protomer by two flexible loops and a four-helix bundle. The equivalent structural elements were shown to mediate relative motions of the ATG9A protomers between the state A and B conformers. For consistency with nomenclature adopted for other ATG9 domain proteins we refer collectively to these structural features of ATG9B as the HINGE region (Figure 4A) [9,36]. The degree of structural homology between the structure of ATG9B and those of ATG9A is very high overall, except for the region comprising TMHs 3 and 4. Thus, structural alignments of ATG9B monomers resulted in Cα root mean square deviation (RMSD) of 2.97 Å across 453 pairs with ATG9A in state A (PDB ID 6WQZ; Figure 4B), 3.30 Å across 448 residues pairs with ATG9A in state B (PDB ID 6WR4; Figure 4C), and 3.38 Å across 457 residue pairs with ATG9A in lipid nanodiscs (PDB ID 7JLP); Figure 4D). By contrast, excluding TMHs 3 and 4 from the alignments decreased the RMSDs to 1.15 Å over 315 residue pairs, 1.11 Å over 304 residue pairs, and 1.10 Å over 345 residue pairs, respectively. We note that the relative positions of TMHs 3 and 4 in ATG9B were found to be intermediate to the one observed in the ATG9A structures (Figures 4B–D).

Multibody refinement [38] of ATG9B micelles, where each subunit of the homotrimer is defined as a rigid entity, revealed conformational heterogeneity in the relative orientations of the protomers in the cryo-EM data. Approximately 88% of the variance in the particle set could be accounted for by 9 eigen vectors/principal components (Figure S2A). Motion along vectors 1 (~13% of variance), 4 (~10% of variance) and 7 (~8% of variance) were chosen for visualization as these three illustrate the three types of relative motion between the protomers represented by the 9 vectors (Movie 1). ATG9B particles, particularly along eigen vector 4, occupy a continuous distribution of conformational states that lie in-between what would be the equivalent of ATG9A state A and B. The observation that the diameters of the central and perpendicular pores are not constant but are rather dynamic may have functional implications (Movie 1).

Comparison of the globally refined consensus ATG9B structure with the models generated using AlphaFold (AF) [39] (predicted Local Distance Difference Test/pLDDT >50) was also insightful with respect to the conformational dynamics of the protein (Figure 4E and S2B). Structural alignment of the entire protomers resulted in a Cα RMSD of 2.37 Å across 461 residue pairs and 0.98 Å across 377 residues pairs excluding TMHs 3 and 4, because these helices are in a closed state B-like conformation in the AF model (Figure 4D). Another striking difference between the experimental and AF predictions of ATG9B are the C-terminal α helices (CTHs) downstream of the ATG9 domain (185–676) specifically 703–714 (CTH1), 721–746 (CTH2), 764–773 (CTH3), 789–805 (CTH4), and 823–848 (CTH5) forming a helix bundle above the HINGE region creating a C-terminal platform (Figure 1C). AF similarly predicts a C-terminal platform in ATG9A: CTH1 and 2 of ATG9A have been visualized experimentally in the state A structure and crosslinking mass spectrometry has shown that the C terminus beyond the ATG9 domain folds back to interact with the domain, supporting the AF predictions (Figure 4B,D and S2C) [8,36]. The C-terminal platforms of both ATG9B and ATG9A occlude the cytosolic openings of the central pore and perpendicular branches (Figure S2D); the conservation of their sequence suggests that they have a functional role (Figure 1C).

### ATG9B lipid binding and scramblase activity

ATG9A and *Saccharomyces cerevisiae* Atg9 were demonstrated to be lipid scramblases, and lipid binding to the lateral branch (Site I) and the vertical opening of the central pore (Site II) have been implicated in this activity [6,9]. To determine whether these lipid binding sites were preserved in ATG9B, we applied Consurf and Jalview conservation analyses in combination with lipid docking using Swiss Dock [40]. In total, 23 putative lipid interacting residues at both sites of ATG9B were identified based on equivalence to ATG9A residues found in proximity (<5 Å) to observed lipids in the nanodisc structure (PDB ID 7JLP) [6]. Of these, 5 were identified as putative lipid head group interacting and 18 as acyl chain interacting residues (Table 3). The head group interacting residues were either charged or polar (Arg146, Tyr249, Glu69, Tyr471, Ser423 in ATG9A) and thus capable of forming electrostatic interactions with the hydrophilic lipid head. Of the 23 residues, twelve are fully conserved among Chordata ATG9s and a further 6 are replaced by an amino acid with the same physicochemical properties (Table 3). Among the head group interacting residues Arg146 and Tyr249 of ATG9A are substituted to Gln295 and Leu398 in ATG9B, respectively. The position of the acyl chain interacting residue Leu373 of ATG9A is occupied by a hydrophobic residue, except in the ATG9B orthologs from primates, rodents, lagomorphs and shrews (Euarchontoglires), that feature an Arg residue in the equivalent positions (corresponding to Arg524 in humanATG9B). Another taxon-specific difference is Ser423 in ATG9A, which is fully conserved across species and in both paralogs except in marsupial ATG9B, where there is a cysteine residue instead.

**Table 3.** Lipid binding site analysis.

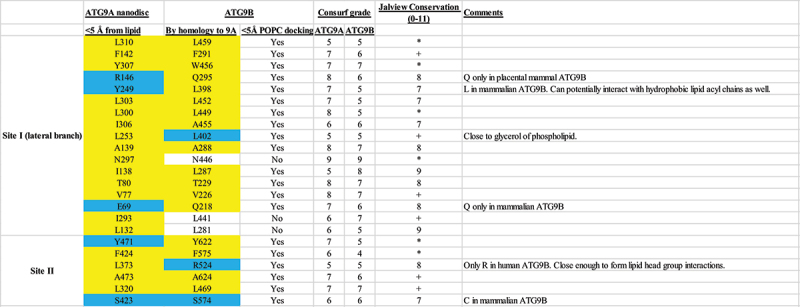

Residues interacting with POPC head group are highlighted in yellow and those interacting with POPC acyl chains are highlighted in blue. Consurf grade indicates conservation (9) or variability (1) at this position in the sequence. Conserved columns in the MSA are indicated by ”*‘ (score of 11 with default amino acid property grouping), and columns with mutations where all properties are conserved are marked with a ’+” (score of 10, indicating all properties are conserved).

**Figure 5.**
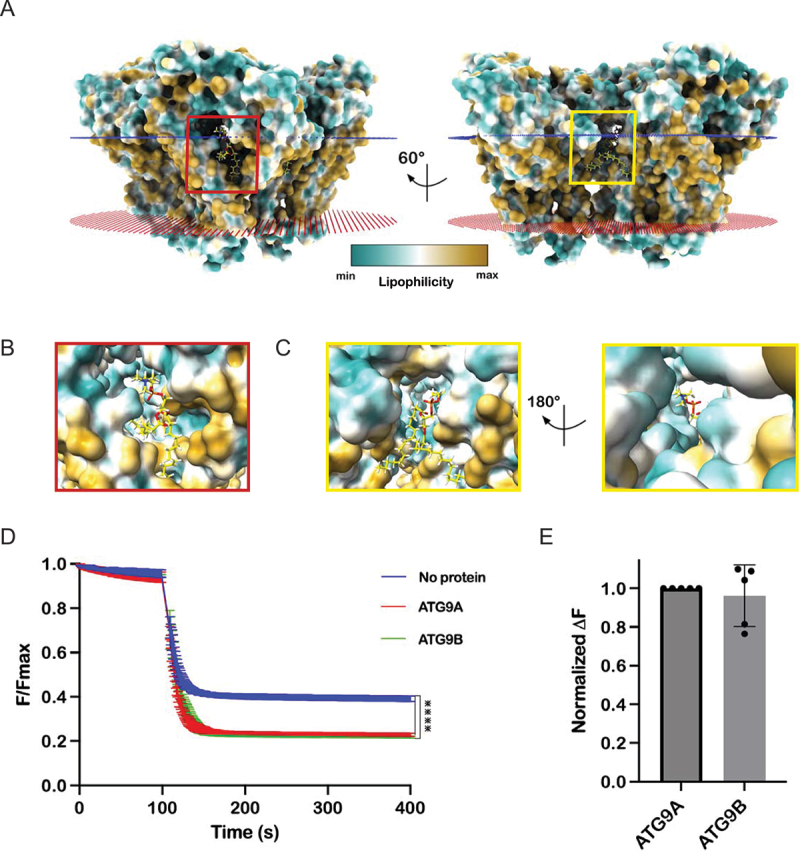
Lipid docking suggests ATG9B is a functional lipid scramblase. (A) POPC (yellow sticks) docked at putative lipid bindings Site I (red box) and Site II (yellow box). Structure of ATG9B shown as surface colored by lipophilicity/hydrophobicity. Close up of POPC in Site I (B) and Site II (C). *in vitro* scramblase assay activity of ATG9B and ATG9A in proteoliposomes. Error bars are standard deviation (SD) (*n* = 3), statistical analysis was done using one-way Kruskal-Wallis test (****; *p* < 0.0001). Data were fit to a ‘plateau followed exponential decay function with R^2^ values of 0.9988, 0.9965 and 0.9743 for the control, ATG9A and ATG9B data respectively (D). (E) quantification of change of fluorescence in ATG9A vs ATG9B at 200 seconds, normalized to ATG9A, error bars are SD (*n* = 5), statistical analysis done by unpaired t-test.

Using *in silico* methods, we docked palmitoyl-2-oleoyl-sn-glycero-3-phosphocholine (POPC) onto the lateral branch (Site I) and vertical opening of the central pore (Site II) of the ATG9B structure (Figure S3). This lipid was found bound at these sites within ATG9A homotrimer embedded in lipid nanodiscs, so this structure could be used to validate the *in silico* docking results. The docking poses for POPC at Sites I and II of ATG9B that were most similar to, and in the case of Site I was also the top scoring pose in the docking, were chosen for analysis. Twenty of the 23 predicted lipid interacting residues were < 5 Å of the docked lipid (Figure 5A - C; Table 3). There were several plausible docking poses predicted at each site and interacting residues besides those identified by correspondence to ATG9A were also predicted, suggesting flexibility in the protein-lipid interaction at these sites and potential differences between the paralogs.

The favorable docking of POPC and the high conservation of the identified putative lipid interacting residues suggested that ATG9B is a lipid scramblase. To test this hypothesis, we reconstituted purified ATG9B and ATG9A in liposomes doped with fluorescent lipid, 1,2-dioleoyl-sn-glycero-3-phosphoethanolamine-N-(7-nitro-2–1,3-benzoxadiazol-4-yl ammonium salt) (NBD-PE). To monitor potential scrambling activity, we measured the quenching of NBD fluorescence upon the addition of dithionite. For both ATG9B and ATG9A, the NBD fluorescence was reduced to a greater extent than in control (without protein) that is ~ 22%, ~23% and ~ 41% of the maximum fluorescence respectively, consistent with the ATG9 proteins mediating equilibration of NBD-PE between the bilayer leaflets of the liposomes (Figure 5D). These data could be fit by least squares regression to a “plateau followed by one phase exponential decay” function with R^2^ values of 0.9988, 0.9965 and 0.9743 for the control, ATG9A and ATG9B data respectively, indicating that the kinetics of NBD-PE chemical reduction by dithionite and scrambling are indistinguishable by this assay [41]. To compare ATG9A and ATG9B scramblase activity, we analyzed the normalized change in fluorescence at a time point after which the NBD-PE in the outer leaflet had been reduced in the control samples, as previously described [6]. No significant difference was detected between the activities of the two paralogs by this assay, confirming that ATG9B is an active lipid scramblase similar to ATG9A (Figure 5D – E).

### ATG9B can function in the autophagy pathway

**Figure 6.**
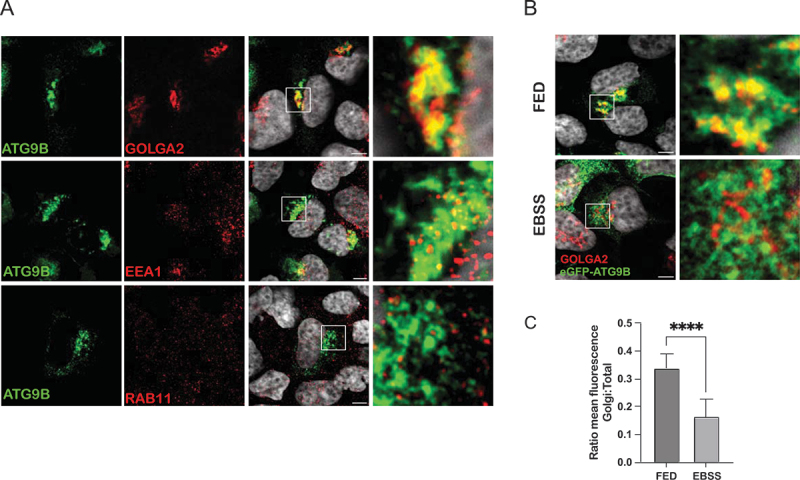
ATG9B intracellular localization and dispersal upon starvation. (A) Representative images of immunofluorescence experiments in HEK293A cells transiently over-expressing eGFP-ATG9B and stained for GOLGA2/GM130 as a Golgi marker, EEA1 as an early endosomal marker and RAB11 as a recycling endosomal marker. (B) Representative images of immunofluorescence experiments in HEK293A cells transiently over-expressing eGFP-ATG9B and stained for GOLGA2 as Golgi marker in fed or 2 h EBSS starvation conditions. Merge of green (eGFP-ATG9B) and red (GOLGA2) is shown. (C) quantification from 3 independent experiments of ATG9B dispersal as ratio between Golgi:total fluorescence comparing fed/starvation conditions. Error bars are SD. Statistical analysis was done using t-test; ****, *p* ≤ 0.001. Scale bar: 5 µm.

**Figure 7.**
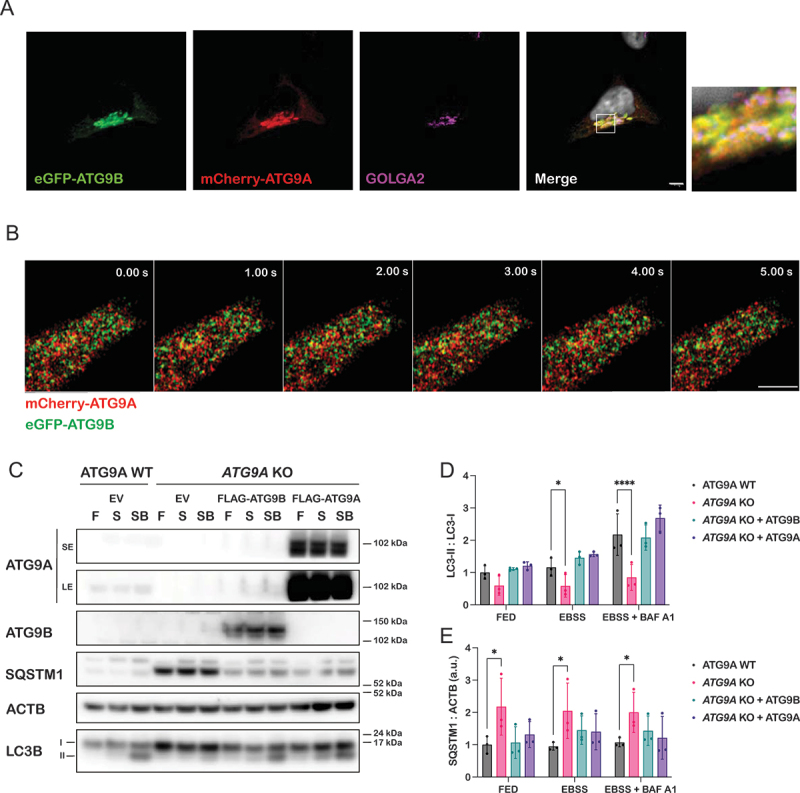
ATG9B is functional in autophagy. (A) Representative images of immunofluorescence experiments in HEK293A cells transiently over-expressing eGFP-ATG9B, mCherry-ATG9A, stained for GOLGA2/GM130 as Golgi marker. Scale bar: 5 µm. (B) montage of live cell imaging of mCherry-ATG9B and eGFP-ATG9A. Scale bar: 5 µm. (C) autophagy flux rescue experiments in ATG9A WT or *ATG9A* KO cells overexpressing FLAG-ATG9B or FLAG-ATG9A for 24 h. Cells were cultured in full medium (F) or either with EBSS (S) or EBSS supplemented with 100 nM bafilomycin A_1_ (SB) for 2 h and then processed by western blot. Indicated antibodies were used. Quantification of (D) LC3 lipidation and (E) SQSTM1 from three independent experiments. Error bars correspond to SD and dots represent each experiment normalised to ATG9A WT control. Statistical analysis was done using one-way ANOVA; *, *P* ≤ 0.05; ****, *P* ≤ 0.001.

The high level of homology shared by the core domains of ATG9B and ATG9A, as well as the ability of ATG9B to scramble lipids *in vitro* led us to hypothesize that ATG9B could also be functional for autophagy. Indeed, ATG9B was previously shown to play a role in autophagy [23,24,26], however its ability to substitute for ATG9A function has not been tested. Since the trafficking of ATG9A between different membrane compartments is critical for its role in autophagy, we investigated the intracellular localization of ATG9B. We expressed human ATG9B with an N-terminal GFP tag as we were unable to produce or obtain a specific antibody to detect ATG9B in cells by immunofluorescence. Like ATG9A, ATG9B was mainly co-localized with the Golgi marker GOLGA2/GM130, to a lesser extent with early endosomes, positive for EEA1, and recycling endosomes labeled using RAB11 (Figure 6A). ATG9A dispersal upon nutrient starvation is required for its function during autophagosome biogenesis [15]. Concordantly, ATG9B dispersed upon starvation, compared to “fed” conditions, and lost its co-localization with the Golgi marker GOLGA2 (Figure 6B,C). Additionally, in HEK293A cells transiently overexpressing mCherry-ATG9A and eGFP-ATG9B the two proteins mainly colocalized in the Golgi region, as expected (Figure 7A). However, live imaging experiments revealed that although the vesicular compartment of ATG9A and ATG9B partially overlap they were also present in distinct vesicular pools, which may indicate different biological functions (Figure 7B, Movie 2).

We next tested whether ATG9B was involved in the autophagy pathway. To this aim, autophagy flux was analyzed in cells where endogenous ATG9A was genetically knocked out (*ATG9A* KO cells) and rescued by exogenously expressing either 3×FLAG-6×His-ATG9A or 3×FLAG-6×His-ATG9B (Figure 7C). Compared to wild type cells, *ATG9A* KO cells failed to engage autophagy upon starvation, as shown by the accumulation of the autophagy receptor SQSTM1/p62 (~2 fold, *p* value = 0.021) and the decrease of LC3B lipidation (LC3B-II), which was reduced by at least 2 fold under nutrient starvation alone (*p* value = 0.042) (Figure 7D) and when combined with bafilomycin A_1_ treatment (*p* value < 0.0001) (Figure 7E). Overexpression of FLAG-tagged ATG9B restored the autophagic flux to a similar extent as ATG9A overexpression, as measured by the increase in LC3-II upon starvation and Bafilomycin A1, as well as the degradation of SQSTM1 upon starvation-induced autophagy (Figure 7C–E). These results support our conclusion that ATG9B shares similar membrane compartments as ATG9A, disperses upon nutrient starvation and is functional for autophagy.

### ATG9B can form a complex with ATG2A

**Figure 8.**
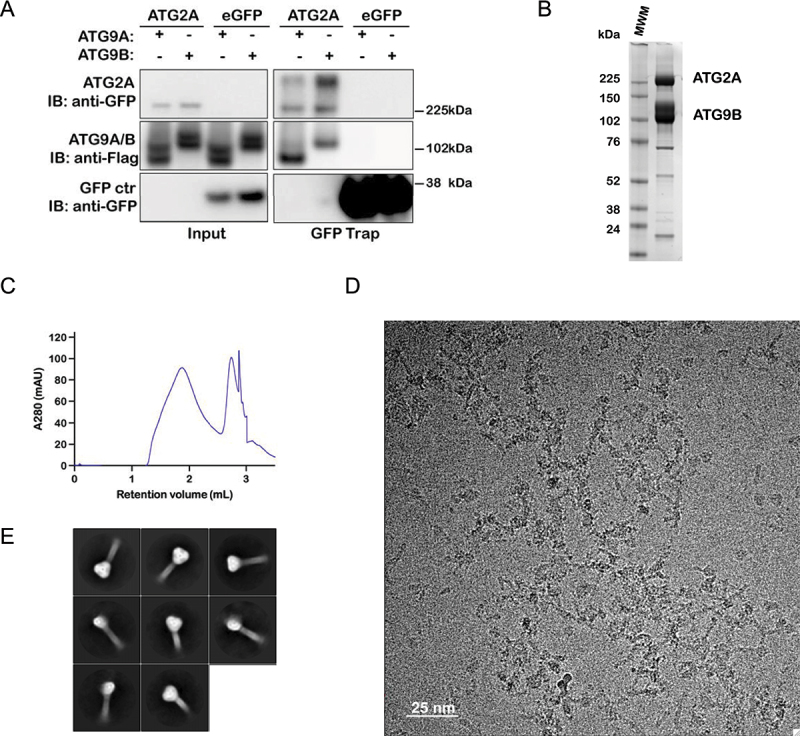
ATG9B forms stable complex with ATG2A. (A) co-immunoprecipitation by GFP-TRAP of 3×FLAG-His_6_-TEV-ATG9B or 3×FLAG-His_6_-TEV-ATG9A by eGFP-ATG2A from cell lysates. Representative experiment of *n* = 3 (B) Coomassie Brilliant Blue stained gel of co-purified ATG9B and ATG2A and (C) SEC of this sample. (D) Representative cryo-EM micrograph of ATG9B-2A sample and (E) 2D class averages from these data.

ATG9A forms a complex with ATG2A to facilitate lipid delivery during autophagosome biogenesis. ATG9B also interacts with ATG2A as demonstrated by their co-immunoprecipitation (Figure 8A). To determine whether the interaction was direct, we purified the ATG9B-ATG2A complex (Figure 8B) and imaged it by cryo-EM. Microscopy revealed particles with rod-like ATG2A and globular ATG9B domain (Figure 8C,D). 2D class averages of particles from these data revealed crotchet shaped particles similar to those reported for the ATG9A-ATG2A complex indicating that ATG9B and ATG2A can interact directly and form a heterotetrametric complex (Figure 8E). As was the case for the ATG9A-ATG2A complex, a reliable 3D reconstruction of the ATG9B-ATG2A complex could not be achieved likely due to flexibility of ATG2A, for which the is no high-resolution structure despite several attempts [5,8,42]. Taken together, our results show that ATG9B can take the place of ATG9A in autophagy and likely does so by a similar molecular mechanism involving lipid delivery to the nascent phagophore.

**Movie 1**. Conformational dynamics of ATG9B along eigen vectors 1, 4 and 7 of multibody analysis of cryo-EM data.

Movie 2. Live cell imaging of eGFP-ATG9B and mCherry-ATG9A.

Movies can be found via the preview link below https://data.mendeley.com/datasets/sxnyrhycff/2

## Discussion

According to our molecular phylogenetic analysis, the emergence of ATG9A and ATG9B was likely the result of the genome duplication that gave rise to jawed vertebrates (gnathostomes). In mammals, the ATG9 domain of ATG9B appears to be undergoing rapid evolution. By contrast, the ATG9 domain of ATG9A seems to be under more stringent selection pressures, presumably conserving more of the functions of the parental gene (Figure 1). It remains to be explored whether the greater divergence observed for the ATG9 domain of mammalian ATG9B is a result of positive selection driven by the development of a new function, or of a relaxation of functional constrains due to gene redundancy. Others have suggested that ATG9B may have acquired novel functions during placental evolution [21,22,43]. The ATG9 domain is conserved across eukaryotes supporting a common molecular structure and function. We showed that ATG9B forms homotrimers maintained by extensive interactions in the membrane via domain swapping of TMHs 3 and 4 with the adjacent protomer interlocking them together (Figure 3).

The conformation of our consensus structure is in a transition state between the open (state A) and closed (state B) conformers reported for ATG9A [36]. Multibody analysis of ATG9B particles used in the consensus refinement of the structure revealed that the protomers are flexibly orientated relative to each other, occupying a continuous distribution of conformational states, including but not limited to the open and closed states described for ATG9A (Figure 4, Movie 1). By extension, both the diameter of the perpendicular branch, which has been suggested to provide access to lipids from the cytosolic side through ATG2A, and the central pore, which has been demonstrated to play a role in lipid scrambling, are dynamic, effectively dilating and contracting (Movie 1) [6,8,36].

Considerable conformational variability was anticipated by MD of the yeast Atg9 structure in lipid membrane [9]. The Atg9 structure was determined in an open state A-like conformation which transitioned to a closed state B-like conformation during the simulation reducing exposure of the hydrophobic bilayer core to the solvated central pore. These results suggested potential mechanisms for modulating these conformational dynamics specifically through changes in the physical properties of the lipid bilayer, such as lateral pressure and rigidity, which can in turn be modulated by the lipid composition of the membrane [44,45].

Given the conservation of the ATG9 domain across eukaryotes, its conformational dynamics are likely conserved and play a role in the molecular functions of the ATG9 proteins. A potential role for the state A/B-like conformational dynamics in the lipid scrambling mechanism has been proposed [6,9]. Recently, a mutant of yeast Atg9, Atg9^F627^ that shown to be unable to rescue autophagy flux but did not affect scramblase or lipid transfer activity *in vitro* [46]. It was suggested, based on the functional characterization of this yeast mutant and the human ATG9A^F382A^ (F533 in human ATG9B) mutant in TMH3, that the trimerization interfaces of the protein may play a role beyond lipid delivery and this change at these interfaces may be the cause of the defective autophagy flux. Based on the structural analysis reported here we suggest that the proposed disruption of the trimerization interfaces may impair the conformational dynamics described for Atg9 and ATG9A [6,9] and here for ATG9B.

Sequence conservation of putative lipid binding sites in ATG9A and ATG9B and lipid docking suggested that ATG9B is a functional lipid scramblase, which we have confirmed for ATG9B using an *in vitro* scramblase assay (Figure 5). Although most of the putative lipid interacting residues are conserved between ATG9B and ATG9A, some residues have diverged in the mammalian ATG9B orthologs suggesting that there might be differences in lipid species preference. However, the measured scramblase activity of ATG9B and ATG9A were quantitatively indistinguishable in the liposomes used here (Figure 5). It has been suggested that these scramblase assays, such as the one used here, may not be sensitive enough to detect subtle differences between proteins and substrates and thus may not recapitulate the cellular conditions necessary to bring such subtleties to the fore [46]. It does appear however, if only qualitatively, that the scramblase activity is similar to that of ATG9A in the context of macroautophagy. Along the same lines the results of the cell-based experiments would support a conservation of this function, but we cannot exclude that the rescue is also due to other conserved functional properties of ATG9A and ATG9B (Figure 7).

Due to the different lengths of the *N*- and C-termini in ATG9A and ATG9B, these regions may confer distinguishing activities to the two paralogs. We note that the N-terminal region of most mammalian ATG9B orthologs contains a stretch of 6 proline residues (29-PMPLPPPPPP-38 in human). Translation of poly-proline sequences, in particular those with more than 3 prolines, are typically thought to contribute to ribosome stalling and require the translation elongation factor EIF5A [47]. Furthermore, the 5’ sequence of the canonical isoform 1 (Q674R7–1) lacks a defined Kozak consensus site. Thus, the translation of the full-length protein encoded by the canonical isoform may be highly regulated and tissues expressing ATG9B may preferentially use EIF5A resulting in the translation of a protein corresponding to the canonical isoform 1. Alternatively, it is possible that a start site downstream of the predicted start codon may be the initiating codon, for example Met105, which has a Kozak sequence. This hypothesis remains to be tested.

The low homology of the *N*- and C- termini suggests that potential interactions mediated by these regions and their regulation may differ between the ATG9A and ATG9B. Interestingly, the ATG13-ATG101 interacting HDIR in the C terminus of ATG9A [33,34], is not conserved in ATG9B. Based on this ATG9B may interact differently or not at all with ATG13 and ATG101. The ATG9A-ATG13-ATG101 interaction has been shown to play a role in mitophagy by recruiting the ULK1 complex and increasing the efficiency of ATG2A lipid transfer activity as part of a ATG9A-ATG2A-ATG13-ATG101 complex [33,34]. Given that age-related decrease in mouse ATG9B expression has been linked to accumulation of aberrant mitochondria in heart muscle [24], it would be interesting to explore if ATG9B is involved in mitophagy and if so, how this mechanism differs from that described for ATG9A given the absence of the HDIR region. Investigating the effect on ATG9B-ATG2A interaction on lipid transfer especially compared to the ATG9A-ATG2A-ATG13-ATG101 complex or the ATG9B-containing complex, if it exists, may also be insightful for understanding the potential specialization of ATG9B. Another notable difference in the termini of the paralogs is the absence of the ULK1 phosphosite (Ser14 in ATG9A) in the N terminus of ATG9B. Phosphorylation of Ser14 has been shown to stimulate increased ATG9A interaction with AP1 and AP2 in response to nutrient starvation promoting the budding of ATG9A vesicles from the trans-Golgi network which is required for autophagy [32]. We show that ATG9B, like ATG9A localizes to the trans-Golgi network and disperses from this compartment upon nutrient starvation however, it remains to be tested if this ATG9B dispersal is regulated by ULK1 or ULK1 phosphorylation of ATG9B. Our live cell imaging of both ATG9A and ATG9B shows that the two proteins partially co-localize in vesicular-tubular compartments. However, further experiments are needed to fully explore ATG9B trafficking, and to determine if it is functionally redundant or is involved in different intracellular functions from those described for its paralog (Figure 6, Movie 2).

In conclusion, our study demonstrates the conserved molecular structure and scramblase activity of ATG9B and ATG9A providing solid ground and context for the interpretation of further work exploring the potential specialized role of ATG9B, in normal and aberrant physiology. We reveal insights into the conformational dynamics of the protein which has implications for understanding the detailed molecular mechanisms of lipid scrambling and potentially other novel functions.

## Materials and Methods

### Proteins sequence analysis and molecular phylogenetic analysis

Sequences were selected manually from Ensembl and NCBI making an effort to cover a wide variety of Chordata taxon [48,49]. MUSCLE and MAFFT were used to calculate the MSA [50,51]. Inspection and edition of MSA was done using Jalview [52]. For phylogenetic calculations, alignments were trimmed to comprise only the ATG9 domain as defined by InterPro/PFAM [53]. Phylogeny was calculated with IQ-tree 1.6.11 using 100 standard bootstrap replicates [54]. The best-fit model selected by Modelfinder was JTT+I+G4 [55]. Trees were visualized and prepared with Dendroscope [56]. To analyze divergence in the flexible regions of ATG9B we used the two separate ortholog MSA as provided by Ensembl Compara, removed incomplete sequences and sequences from invertebrates, and obtained Shannon entropy (H) scores for each position in the individual alignments using the Protein Variability Server [27]. Results were visualized using ggplot2_3.4.2 (https://cran.r-project.org/web/packages/ggplot2/citation.html). The raw MSAs and tree can be accessed on Mendeley data (https://data.mendeley.com/datasets/sxnyrhycff/2).

### Cell lines and cell culture

HEK293A cell lines were obtained from Cell Services at The Francis Crick Institute. Cells were grown in DMEM high glucose (Sigma, D6429) supplemented with 10% heat-inactivated fetal bovine serum (Gibco 10,270–106). Cells were incubated at 37°C, 10% CO_2_ and 90–95% of relative humidity. Specific experimental conditions are indicated in figure legends. Expi293 cells (ThermoFisher Scientific, A14527) were cultured in suspension in Expi293 expression medium (ThermoFisher Scientific, A1435101) at 8% CO_2_, 37°C. *ATG9A* KO cells (Cell services at The Francis Crick Institute) were generated by CRISPR-Cas9 as described in [8].

### Plasmids and DNA transfection

DNA transfection for experiments in HEK293A was performed following the manufacturer’s instructions using Lipofectamine 2000 (Invitrogen 11,668–019) in 1:5 Opti-MEM:DMEM medium (Gibco 31,985–047). For protein production in Expi293 cells were transfected using polyethyleneimine (Polysciences 23,966–1) as described in [8]. A ratio of 3:1, polyethyleneimine: plasmid DNA, was used for transfecting 3×FLAG-6×His-TEV-ATG9A (Genscript), 3×FLAG-ATG2A [8] or 3×FLAG-6×His-TEV-ATG9B (Genscript) in all cloned separately into pcDNA3.1(+) vector (Table 4).

**Table 4.** Antibodiees and plasmids.

Antigen	Source	Dilution	Use
**Antibodies**			
ATG9A	CRUK (in-house)	1/1000	WB
ATG9B	Abcam, ab117591	1/1000	WB
SQSTM1	Cell Signalling Technology 39,749	1/2000	WB
ACTB	Abcam, ab8227	1/2000	WB
LC3B	Abcam, ab48394	1/2000	WB
GFP	CRUK (in-house)	1/1000	WB
FLAG	Sigma-Aldrich, F3165	1/1000	WB
EEA1	BD Biosciences 610,456	1/500	IF
GOLGA2/GM130	BD Biosciences 610,823	1/1000	IF
RAB11	Abcam, ab228924	1/400	IF
LAMP1	BD Pharmigen 555,798	1/400	IF
Plasmid	Origin	Use	
pcDNA3.1-3×FLAG-6×His-TEV-ATG9A	Genescript	Protein production/Autophagy rescue experiments/co-IP	
pcDNA3.1-3×FLAG-6×His-TEV-ATG9B	Genescript	Protein production/Autophagy rescue experiments/co-IP	
pcDNA3.1-mCherry-3×FLAG-6×His-TEV-ATG9A	Genescript	Live imaging experiments	
pcDNA3.1-eGFP-3×FLAG-6×His-TEV-ATG9B	Genescript	Live imaging experiments	
pcDNA3.1-eGFP-ATG2A	In house	co-IP	
pcDNA3.1-3×FLAG-ATG2A	In house	Protein production	

### Protein purification

Expi293 cells in suspension at 3 - 4 × 10^6^ cells/mL were transfected with 3×FLAG-6×His-TEV-ATG9B in pcDNA3.1(+) using polyethyleneimine and harvested after 72 h by centrifugation at 500 *g* for 10 min. Buffers used at various stages in the ATG9B purification were made in 50 mM Tris-HCl, pH 8.5, 200 mM NaCl, 1 mM TCEP (Merck 646,547), 10% glycerol (base buffer). Cells were resuspended in 2.5 mL of base buffer per g supplemented with 2× cOmplete protease inhibitor tablets (Roche 0,505,648,900). An equal volume of 1.8% w/v n-dodecyl-β-D-maltoside and CHS (cholesteryl hemisuccinate) mixed at a ratio of 5:1 in base buffer (Genron, NB-39 -00,015-1 g, and CH210-1 GM, respectively) was added to lyse the resuspended cells and solubilize ATG9B. After incubation at 4°C with agitation the lysate was centrifuged at for 20 min at 4000 *g* and the supernatant incubated with anti-DYKDDDDK G1 affinity resin (Genscript, L00432), using 167 µl resin slurry per g of cells, for 2 h at 4°C with agitation. The resin was removed from the lysate by centrifugation at 1,000 *g* for 2 min at 4°C and the resin was washed 5 times with a volume of wash buffer (20 mM Tris-HCl, pH 8.5, 500 mM NaCl, 1 mM TCEP, 10% glycerol supplemented with 0.001% w/v LMNG [Genron, NG310-25 GM] and 0.0002%w/v CHS) equal to the volume of resin added to the lysate. The washed resin was incubated with wash buffer (supplemented with 2.5 mM adenosine 5’-triphosphate [ThermoFisher Scientific, R0441] and 5 mM MgCl_2_) overnight at 4°C with agitation. The resin was washed 5 times with 0.001%w/v LMNG-CHS (5:1) in base buffer (elution buffer). ATG9B was eluted from the resin with 3×FLAG peptide (Peptide Chemistry STP, The Francis Crick Institute) at 240 µg/mL in the same buffer for 20 min. The elution was repeated twice without the 20-min incubation. The eluted fractions were pooled and concentrated to 100 µl before loading on a superose 6 5/150 GL column (Cytivia 29,091,597). For cryo-grid preparation the column was equilibrated with 20 mM Tris-HCl, 200 mM NaCl, 0.001% w/v LMNG-CHS (5:1), pH 8.5, otherwise, elution buffer was used.

3×FLAG-6×His-TEV-ATG9A in pcDNA3.1(+) and 3×FLAG-ATG2A in pcDNA3.1(+) were expressed as above in Expi293 cells. ATG9A was purified as previously described [8]. Briefly ATG9A-expressing cells were resuspended and lysed in 1.2% LMNG-CHS in base buffer without 10% glycerol by agitation at 4°C for 40 min. The lysate was centrifuged at 4000 *g* for 20 min and the supernatant incubated with anti-DYKDDDDK G1 affinity resin for 2 h before washing the resin with base buffer without 10% glycerol supplemented with 0.001% w/v LMNG-CHS (5:1). ATG9A was eluted using 3×FLAG peptide as described above. For purification of the ATG9B-2A complex, cells expressing ATG9B and ATG2A were mixed after resuspension, and the complex purified and analyzed by SEC using the same protocol as described for ATG9B alone.

### Cryo-EM single particle analysis

Four µL of peak SEC fractions of ATG9B and ATG9B-2A at ~0.3 mg/ml were applied to gold C-flat grids (Electron Microscopy Sciences, CF-1.2/1.3-4Au-50) and incubated for 60 s at 100% humidity before blotting for 3 s and plunge freezing in liquid ethane using a Vitribot VI (ThermoFisher Scientific).

ATG9B particles were imaged on a Krios Titan G3i microscope (FEI) operating at 300 keV and equipped with a K2 Summit direct electron detector (Gatan) and a BioQuantum energy filter (Gatan). Initially 17,551 micrograph movies were collected at a calibrated magnification of 46,296, resulting in a pixel size of 1.08 Å at the specimen level, 20 eV energy filter slit, a nominal defocus range −1 to −3 µm, and a total dose of 48 e^−^/Å^2^, fractionated over 32 frames. The micrograph movies were aligned and summed, applying dose weighting as implemented in MotionCor2 [57], and contrast transfer function (CTF) parameters were estimated using Gctf software [58]. Particles, initially picked with crYOLO [59], were subjected to reference-free 2D classification in cryoSPARC [60]. The resulting 2D class averages, low pass filtered to 20 Å, were used as templates to pick all micrographs using Gautomatch (http://www.mrc-lmb.cam.ac.uk/kzhang/). The resulting set of 4,022,224 particles were subjected to several rounds of 2D classification in cryo-SPARC, where junk and poorly defined particles were discarded after each round, yielding 195,921 particles. 3D reconstructions from this particle set displayed anisotropy as assessed by 3DFSC software [61]. To mitigate this pathology, a second dataset of 19,666 micrograph movies was collected using imaging parameters as above, but with a 30° stage tilt. Following motion correction with MotionCor2, 2,796,248 particles were picked. Local CTF estimation for all particles picked on the tilted data was performed using goCTF [62]. After several consecutive rounds of 2D classification in cryoSPARC 42,168 particles remained in well-defined classes. These were combined with the 185,789 particles from the untilted dataset and subjected to 3D classification in RELION-3.1, starting with an *Ab initio* model generated in cryoSPARC. The 3D classification into 3 classes, without imposing symmetry, resulted in a single high-resolution class. The resulting 160,986 particles were used for unbinned 3D refinement, followed by CTF refinement in cryoSPARC and Bayesian polishing in RELION-3.1. The polished particles were subjected to 3D classification in cryoSPARC into two *Ab initio* classes, with maximum resolution set to 8 A, class similarity 0.1, with no symmetry imposed resulting in a single well-defined class comprising 152,410 particles. These were used for another round of *Ab initio* classification to yield the set of 104,050 particles, which were used to generate the final reconstructions using non-uniform refinement in cryoSPARC with and without C3 symmetry imposed. The resolution reported is according to the gold-standard FSC 0.143 criterion [63,64] (Figure S1B and Table 1). As assessed using 3DFSC software [61], the final reconstructions retained moderate anisotropy (Figure S1C), resulting in a 3D FSC sphericity of 0.967. For illustration purposes and to aid model building, the cryo-EM maps were post-processed using DeepEMhancer [35]. To analyze molecular motions, multibody refinement [38,65] was performed in Relion-4.0. During multibody refinement, each of the three protomers, including TMH3 and 4 donated by the neighboring ATG9B chain, was defined as a rigid body.

ATG9B-2A grids were imaged using a Talos Arctica microscope (FEI) operating at 200 keV and equipped with a Falcon III direct electron detector (Gatan). In total, 4,939 micrograph stacks were acquired at a calibrated magnification of 111,111 and a pixel size of 1.26 Å. Data collection was conducted in using the linear integration mode of the Falcon III detector, with a defocus range of −1.0 to −3.5 µm, and a total dose of 82 e^−^/Å^2^ over 10 frames. The micrograph stacks were aligned and summed with dose weighting using MotionCor2, and CTF parameters were estimated using Gctf. The initial set of 810,427 particles picked in crYOLO using the general model, were subjected to reference-free 2D classification in RELION-3.1 [66]. Well-defined 2D class averages were used as templates for auto-picking particles from micrographs using RELION-3.1 resulting in a set of 800,092 particles. These were subjected to several rounds of 2D classification in cryoSPARC and RELION-4.0. Extensive classification efforts notwithstanding, a reliable 3D reconstruction could not be achieved for the ATG9B-2A complex due to flexibility of ATG2A.

### ATG9B atomic model building

A monomer of the AlphaFold model of ATG9B state the number of the model and residues used (predicted Local Distance Difference Test > 50) was rigid body docked into the postprocessed C3 cryo-EM map from cryoSPARC using UCSF Chimera [67] followed by removal of regions that were not supported by the cryo-EM map. Secondary structure elements were fitted into the map using real-space refinement in COOT [68]. This new model was then adjusted residue by residue to better fit the model to the map and while maintaining reasonable protein geometry which was assessed iteratively using MolProbity [69]. The model of the monomer was replicated to produce the trimer and the homo-trimer structure was subjected to real space refinement using Phenix program suite [70] in the cryo-EM map refined imposing C3 symmetry (Figure S1E).

Lipid docking was performed on the monomer using the SwissDock server (http://www.swissdock.ch/). The SMILE representation for 1-Palmitoyl-2-oleoyl-sn-glycero-3-phosphocholine (PubChem ID 5,497,103) was used to generate a 3D model of the lipid in UCSF Chimera and the model was energy minimized before submitting to server for docking. Visualization of docking results was done using UCSF Chimera. Structure alignment and figure preparation were done using USCF Chimera and Chimera X. Conservation analysis was done on the MSA used for the phylogenetic analysis using Consurf [71] and Jalview [52]. The raw MSA used for the conservation analysis can be accessed Mendeley data (https://data.mendeley.com/datasets/sxnyrhycff/2).

### Lipid scramblase assay

POPC, 1,2-dioleoyl-sn-glycero-3-phosphatydil-L-serine and NBD-PE (Avanti Polar Lipids, 850457C,840035C, 850757C) was mixed at a molar ratio of 95.5:5:0.5 in chloroform then dried under N_2_ and for an hour under vacuum. The lipid film was resuspended at room temperature with mixing in 80 mM HEPES, 150 mM NaCl, pH 7.5 (liposome buffer) to a final concentration of 3 mM. The liposomes were subjected to 3 cycles of freeze thaw before extrusion 21 times through a 400-nm pore filter (Avanti polar lipids, Merck 610,007). For proteoliposome reconstitution, purified ATG9B and ATG9A at 5 µM in 20 mM Tris-HCl, pH 8.5, 500 mM NaCl, 1 mM TCEP, 10% glycerol supplemented with 0.001% w:v LMNG were incubated for 1 h with mixing at 4°C, at a final protein concentration of 55 nM, with 654 µM liposome preparation and 0.44% w/v Triton X-100 (Thermofisher Scientific 13,454,259) in scramblase assay buffer (liposome buffer supplemented with TCEP to a final concentration 0.3 mM). Bio-beads were added, and the mixture incubated overnight with agitation at 4°C to adsorb the detergents. For the assay the liposomes were diluted 10.4-fold with assay buffer and NBD-PE fluorescence measured for 100 seconds before reducing it with dithionite (20 µM final concentration).

### Cell lysis and western blotting

For cell lysis, cells were washed three times with ice-cold phosphate-buffered saline (PBS; 137 mM NaCl, 2.7 mM KCl, 8 mM Na_2_HPO_4_, and 2 mM KH_2_PO_4_, pH 7.4), scraped and lysed in TNTE buffer (20 mM Tris-HCl pH 7.4, 5 mM EDTA, 150 mM NaCl, 0.5% Triton X-100) supplemented with protease (Roche, cOmplete EDTA-free 05,056,489,001 and phosphatase inhibitors (Roche, Phostop EASYpack, 04 906 837 001). Cell lysates were centrifuged at 13,000 × g for 10 min at 4°C. Protein concentration was determined using Pierce BCA Protein Assay kit (ThermoFisher Scientific 23,227) following manufacturer’s instructions. Lysates were prepared for electrophoresis in Laemmli SDS-sample buffer and incubated at 65°C for 10 min. Samples were resolved by SDS-PAGE on NuPage 4–12% Bis-Tris Gel (Invitrogen, NP0336) using MES SDS running buffer (Invitrogen, NP0002) and transferred to Immobilon-P transfer membranes (Millipore, IPVH00010). Membranes were blocked with 5% nonfat dry milk (BioRad 1,706,404) in PBS containing 0.1% Tween-20 (PBS-T) for 1 h at room temperature. Incubation of primary antibodies was performed overnight at 4°C in 5% nonfat dry milk or 3.5% BSA (Roche 10,735,086,001). After three washes in PBS-T, membranes were incubated for 1 h at room temperature with secondary antibodies (1:5000) diluted in 5% nonfat milk. Upon incubation, membranes were washed three times with PBS-T and protein detection was performed by using Immobilon Classico Western HRP Substrate (Millipore, WBLUC0500) or Immobilon Crescendo Western HRP Substrate (Millipore, WBLUR0500). Blots were scanned with Amersham ImageQuant 800 (Cytiva). Densitometry analysis of western blots was performed using FIJI (https://fiji.sc/). All the antibodies used in this study are reported with the corresponding working concentrations in Table 4.

### Immunofluorescence and confocal microscopy

Cells were grown on poly-D-lysine treated coverslips to reach 70% confluency the day of the experiments. After the treatments, cells were fixed with 4% paraformaldehyde in PBS supplemented with 0.1 mM CaCl_2_, 0.1 mM MgCl_2_ for 10 min at room temperature. Cells were washed three times with PBS before adding 50 mM NH_4_Cl for 10 min at room temperature and then permeabilized with 50 μg/mL digitonin (Merck Millipore; D141) for 5 min at room temperature. Cells were then washed with PBS and blocked in 5% BSA (in PBS for 30 min at room temperature. Coverslips were incubated upside down with primary antibody in 1% BSA in PBS 1 h at room temperature, then washed three times with PBS and incubated upside down with secondary antibody in 1% BSA in PBS for 1 h. Finally, coverslips were washed three times in PBS and once with deionized water before mounting them on glass microscope slides using 10 µL Mowiol mounting solution (Millipore,475904). Fluorescence images were acquired using a Zeiss LSM 880 Airyscan confocal microscope with Plan-Apochromat 63×/1.4 Oil DIC M27 objective lens. Zeiss ZEN imaging software was used to acquire the images, and, after acquisition, processing was performed using an Airyscan processing tool on the ZEN software provided by Zeiss. All the antibodies used in this study are reported with the corresponding working concentrations in Table S1.

### Live imaging

Cells transfected with eGFP-ATG9B or eGFP-ATG9B/mCherry-ATG9A were seeded on glass-bottom microwell dishes (MatTek Corp., P35G-1.5-14-C) to reach 70% confluency the day of the experiment. Cells were imaged using a Zeiss LSM 880 Airyscan confocal microscope with Plan-Apochromat 63×/1.4 Oil DIC M27 objective lens. Live imaging was performed using Zeiss ZEN imaging software and the acquired movies were processed using an Airyscan processing tool on the ZEN software provided by Zeiss. Plasmids used in this study are listed in Table S1.

### Immunoprecipitation

HEK293A cells overexpressing GFP-ATG2A and 3×FLAG-6×His-ATG9A or 3×FLAG-6×His-ATG9B were lysed on ice in TNTE buffer with protease inhibitors as above. The lysate was centrifuged at 21,000 *g* for 10 min and supernatant incubated with binding control agarose beads (ChromoTek, bab-20) for 1 h at 4°C. The cleared lysate was incubated with GFP-TRAP beads (ChromoTek, Gta-20) overnight at 4°C. The beads were washed four times with TNTE buffer before eluting by incubating with 2.5× Laemmli SDS sample buffer for 5 min at 65°C. Sample were resolved by SDS-PAGE on NuPage 4–12% Bis-Tris Gels before transfer to Immobilon-P transfer membranes. Membranes were blocked before incubating with primary and secondary antibodies as described above. Antibodies used can be found in Table 4.

### Statistical analysis

Statistical tests and analyses were performed using Graphpad Prism 9. The samples sizes, meanings of error bars, type of statistical test performed and the *p* values are detailed in the figure legends and figures.

## Supplementary Material

Supplemental Material
