# Supplementary material for: ATG9B is a tissue-specific homotrimeric lipid scramblase that can compensate for ATG9A
Source: Autophagy. 2023 Nov 8;20(3):557–76. doi: 10.1080/15548627.2023.2275905 (PMC10936676; doi:10.1080/15548627.2023.2275905)
Supplement: Supplemental Material [file KAUP_A_2275905_SM5067.docx]

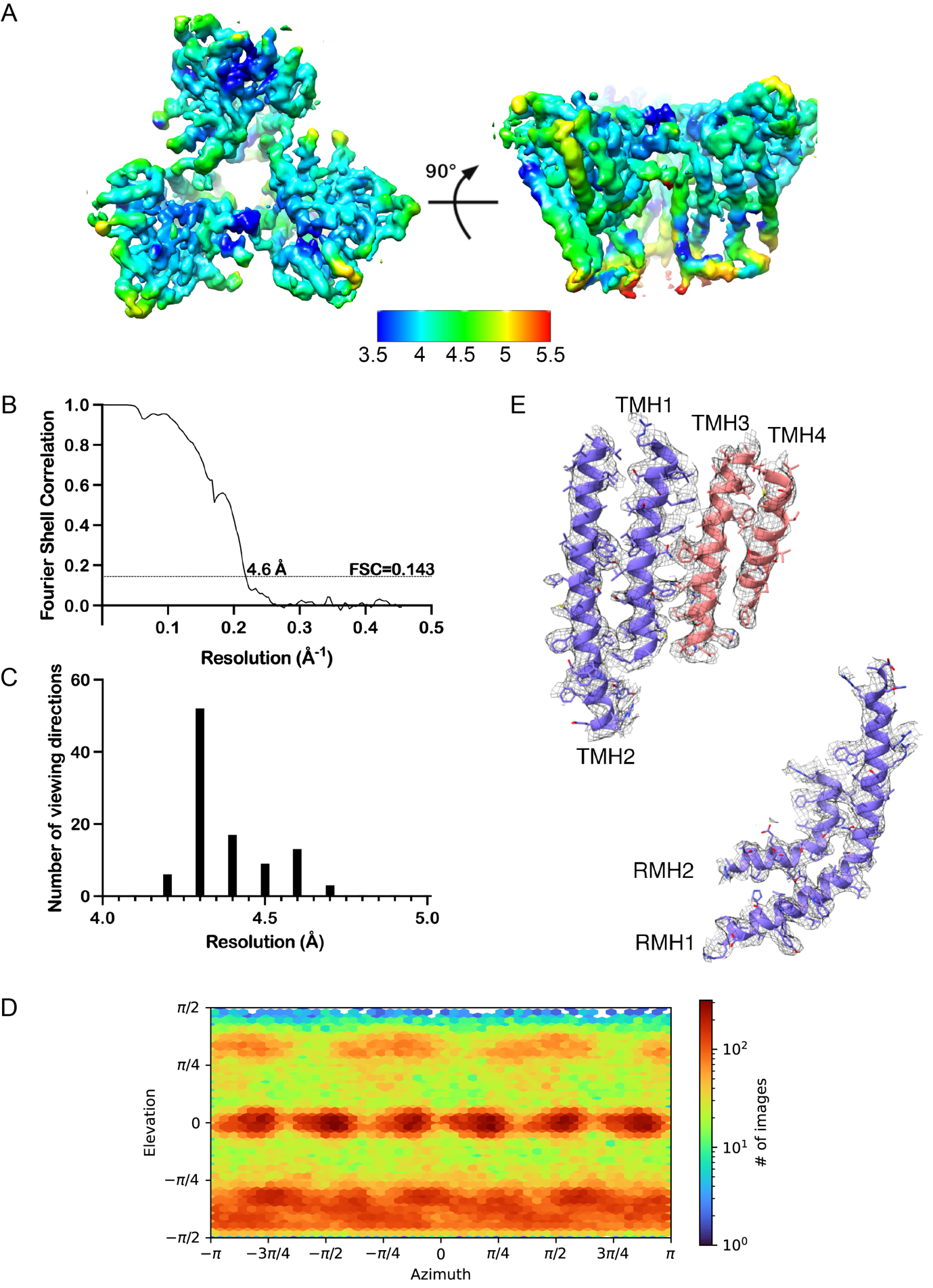


**Figure S1.** Validation of 3D reconstruction of ATG9B from cryo-EM and atomic model building**.** (**A**) C1 3D reconstruction of ATG9B, locally filtered and colored by local resolution (Å). (**B**) FSC curve of 3D reconstruction of ATG9B with no symmetry imposed (C1). (**C**) Histogram of directional FSC values calculated using 3DFSC software (C3 map). (**D**) Particle orientation distribution for particles used in final 3D reconstructions of ATG9B. (**E**) Cryo-EM densities of transmembrane helices of ATG9B (C3 map). RMH: re-entrant membrane helix; TMH: transmembrane helix.


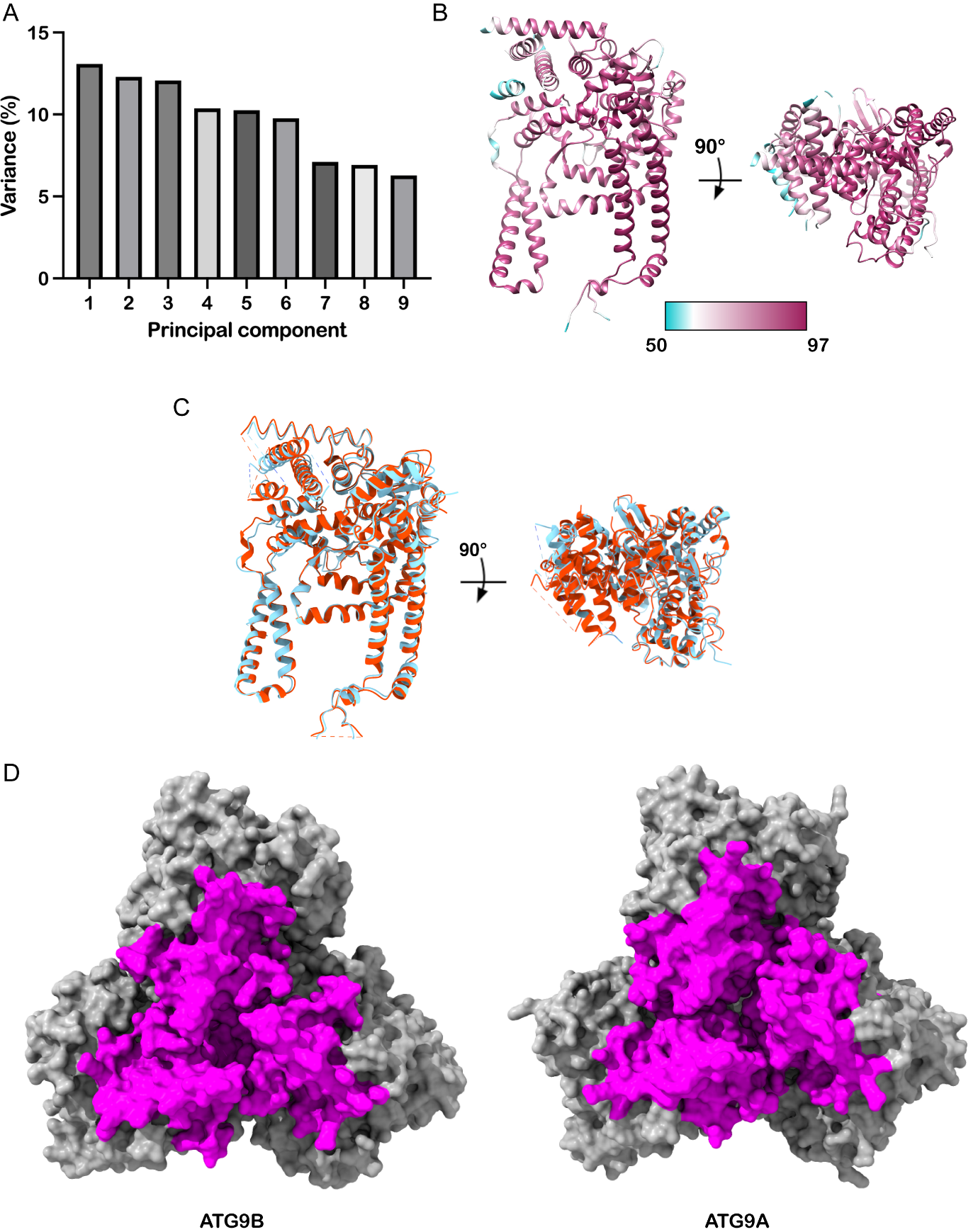


**Figure S2**. Conformational heterogeneity analysis from cryo-EM and Alphafold modelling of ATG9B. (**A**) Principal components of ATG9B and variance from multibody analysis of cryo-EM structure. (**B**) AlphaFold model of ATG9B colored by confidence score (pLDDT>50) (**C**) Alignment of ATG9A (cyan) and ATG9B AF models (pLDDT>50). (**D**) Top views of surface representations AF models of ATG9B (right) and ATG9A (left). C-terminal platforms are colored in magenta.

**Figure S3.** Validation of lipid docking by comparison with lipid conformation observed in nanodisc structure. POPC docking pose (gold sticks) at (**A**) Site I and (**B**) Site II of ATG9B shown in orange red, aligned with ATG9A nanodisc (PDB ID 7JLP; white). POPC from nanodisc structure shown in gray.
